# Deep Machine Learning Model Trade-Offs for Malaria Elimination in Resource-Constrained Locations

**DOI:** 10.3390/bioengineering8110150

**Published:** 2021-10-21

**Authors:** Peter U. Eze, Clement O. Asogwa

**Affiliations:** 1 School of Computing and Information Systems, Faculty of Engineering and IT, University of Melbourne, Melbourne, VIC 3010, Australia; 2 Smart Electronics Systems Research Group, College of Engineering and Science, Victoria University, Melbourne, VIC 3011, Australia

**Keywords:** deep learning, resource optimisation, model quantisation, malaria, digital health, edge devices

## Abstract

The success of deep machine learning (DML) models in gaming and robotics has increased its trial in clinical and public healthcare solutions. In applying DML to healthcare problems, a special challenge of inadequate electrical energy and computing resources exists in regional and developing areas of the world. In this paper, we evaluate and report the computational and predictive performance design trade-offs for four candidate deep learning models that can be deployed for rapid malaria case finding. The goal is to maximise malaria detection accuracy while reducing computing resource and energy consumption. Based on our experimental results using a blood smear malaria test data set, the quantised versions of Basic Convolutional Neural Network (B-CNN) and MobileNetV2 have better malaria detection performance (up to 99% recall), lower memory usage (2MB 8-bit quantised model) and shorter inference time (33–95 microseconds on mobile phones) than VGG-19 fine-tuned and quantised models. Hence, we have implemented MobileNetV2 in our mobile application as it has even a lower memory requirement than B-CNN. This work will help to counter the negative effects of COVID-19 on the previous successes towards global malaria elimination.

## 1. Introduction

The growing accuracy and robustness of deep machine learning models in solving various human problems have increased attempts to also deploy deep learning models in healthcare systems. The applications of deep learning (DL) models in clinical settings include the use of prediction and natural language processing (NLP) algorithms to detect and diagnose diseases. However, in the public health domain, the problem of active case finding and predicting future outbreaks transcends the clinics into the public domain. Health workers and medical researchers have to perform tests and diagnoses outside the clinics and laboratories. This non-static engagement calls for the deployment of mobile health applications that run on edge devices such as mobile phones. These applications, which can also benefit from DL algorithms [1], enable health workers to perform off-clinic diagnosis and case finding.

The DL predictive models deployed in these edge devices often require heavy compute and memory resources [2]. These resources, in turn, consume large amount of energy. Because we use these devices in a mobile scenario, energy conservation becomes important so that these devices do not run out of power before the day’s job ends. Again, in rural areas of developing countries, there is often little or no source of energy to recharge these devices. Hence, there is always a need to optimise the compute, memory and speed of the DL models deployed in mobile health applications for rural case finding.

One of the ways of optimising deep learning models is throughmodel quantisation [2]. Quantisation is the representation of 32-bit floating point (FP32) numbers, which are often used in DL models, with lower bits such as 16-bit floating point (FP16) or 8-bit integer (INT8) [2,3,4]. The effect of quantisation is the reduction in the memory footprint of models by either a factor of 2 or 4 [2,5]. Quantisation reduces both the computational and memory requirement of deep learning models.

Computational optimisation should not significantly affect the prediction or diagnostic accuracy of deep learning algorithms implemented in healthcare systems. Hence, there is a need to assess the trade-offs between computational efficiency and diagnostic accuracy of the quantised models. Sensitivity and Specificity are special parameters used by medical practitioners to establish the performance of a test method. In comparison with data science and statistics, the popular parameters are accuracy, precision and recall [6,7]. Fortunately, recall and sensitivity have the same expression. Specificity can also be easily computed from the confusion matrix generated from a batch machine learning inference. Therefore, in order to satisfy both data scientists and medical practitioners, we utilise accuracy, precision, sensitivity and specificity to measure the predictive performance of different models (and their different quantisation levels) utilised for classification of malaria images in this paper.

**Figure 1:**
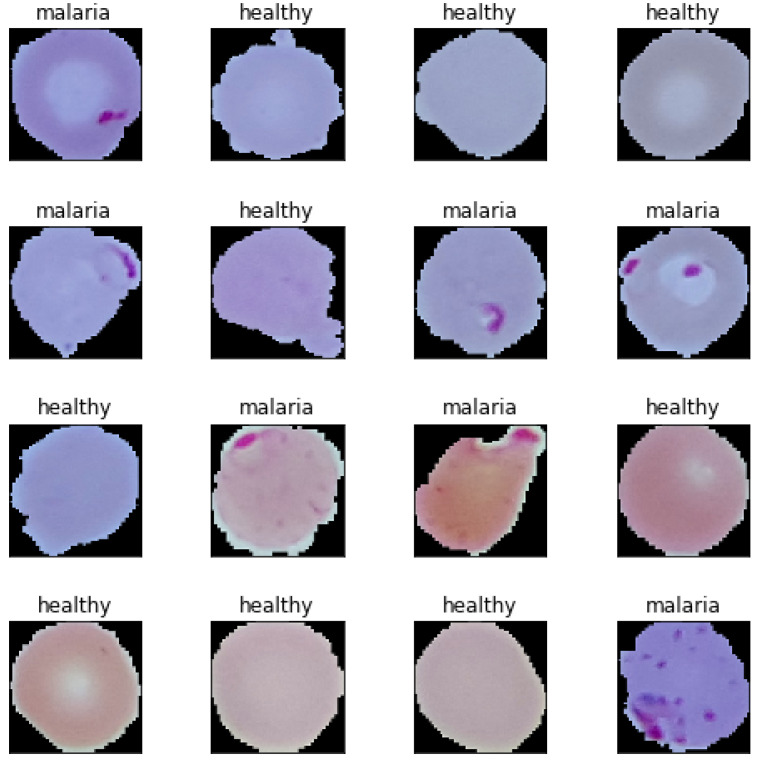
Randomly selected sample dataset [8,10]: Healthy and malaria classes with equal number of images per class.

In this paper, we present the performance (computational and predictive) of four models, each with four different quantisation approaches. The four models are Basic Convolutional Neural Network (B-CNN), a VGG19 based model in which only the last few layers were trained (VGG19-Frozen), a VGG19 with fine-tuned training via image augumentation (VGG19-Finetuned), and MobileNetV2 trained through transfer learning (MobileNetV2). We utilised up to 26,000 malaria-infected and healthy blood smear images [8,9] for training, validation and testing. Some samples of randomly selected images from the data set are shown in Figure 1 for both Healthy and Malaria blood smear image samples.

The aim of this research paper is to systematically select the best model for embedding into a mobile application for the purpose of malaria case finding in regions where malaria has remained endemic.

The rest of this paper is organised; therefore, in Section 2, we highlight the disease burden of malaria and the challenges towards its elimination. In Section 3, we describe the deep machine learning solutions and the design considerations for developing a case finding mobile application in a resource-constrained setting. The results are presented in Section 4 and then discussed in Section 5. We conclude this paper in Section 6. An appendix provides the user interface for the mobile application.

## 2. Challenges of Malaria Eradication in Sub-Saharan Africa

Global efforts to eliminate malaria are thwarted by several factors such as vector transmission, mutation of Plasmodium species causing infection, geographic distribution of vectors and infection, and drug resistance. Eradication of malaria in Africa and other parts of the world has been hampered by both health and technical problems. Other disease burdens, especially the COVID-19 pandemic, have usurped the resources that would have been used for malaria elimination campaigns.

In most cases of malaria, diagnosis microscopy performed on peripheral blood smears remains the most widely used technology. Microscopy has the advantage of detecting all parasite species in the blood sample and can quantify the severity of infection [11]. Nonetheless, microscopy is not a quick diagnostic option in a resource-constrained environment. An efficient malaria microscopy requires highly skilled microscopist available for every diagnosis which are not readily available in resource strained countries. Malaria microscopy requires significant expertise, equipment, electricity, and reagents [12]. An alternative method, the malaria rapid diagnostic test (MRDT), is expensive to run. In addition, there are issues relating to the performance of MRDT in diagnosing pregnancy-associated malaria [13], false positives for a considerable amount of time after malaria treatment [14], and poor understanding of test limitations [15]. As the new world of Artificial intelligence emerge and other digital health solutions are deployed for both clinical and epidemiological solutions, the problem of electrical energy and power problem in developing countries deters sustainable use of these technological solutions. Rapid expansion of diagnostic testing in energy drained regions will require a combination of other diagnostic approaches that are affordable, accessible and less energy intensive. An effective diagnostic device powered by AI can accelerate malaria diagnosis in the aforementioned regions. TensorFlow Lite, for example, works for on-device machine learning with both Android and IOS frameworks. In this paper, we are concerned with demonstrating the design trade-offs among the variables that determine successful deployment of machine learning case finding solution for malaria detection and elimination in energy-scarce countries.

## 3. Methods

An experimental design approach is employed in this research to compare the performance of different deep learning models for malaria detection through blood smear images [10]. We first selected four candidate deep learning architectures and different deep learning methods for comparison. After training the deep learning architectures using the training data, we performed inference with models. Then, we converted the models into a mobile format before applying different levels of post-training quantisation to the weights and activations. For each model, we measured the inference time, test set accuracy, precision and recall. We also recorded the memory size used to store each of the models.

### 3.1. Chosen Deep Learning Architectures and Training Methods

Convolutional neural networks (CNN) are forms of deep learning models that are suited for feature extraction from a structured array of data such as images. These features are patterns such as lines, gradients, contours and shapes. The advantage of deep neural networks such as CNN is that they can use raw input image datasets to extract the features they need. Unlike traditional machine learning algorithms where specific features will be extracted and fed into the training algorithm, CNN extracts the required features themselves.

**Figure 2:**
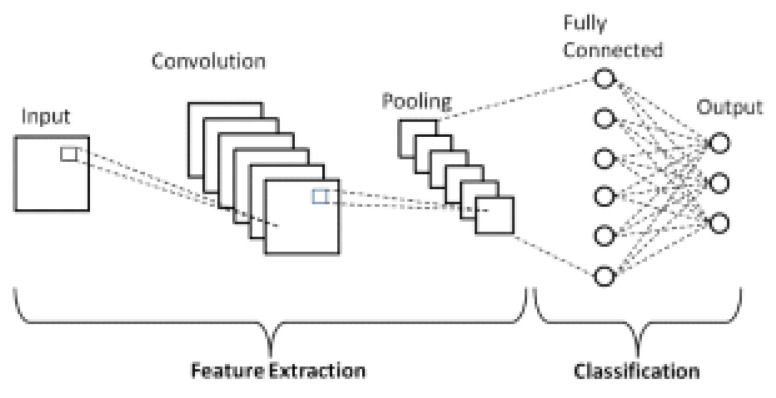
Basic CNN Architecture with three convolution layers alternated with three pooling layers. The output is either malaria or healthy.

The convolutional layers are the powerhouses of a CNN. They are stacks of a network in which each layer is capable of extracting and recognising more complex features. A CNN is made up of 20–30 layers of which convolutional layers form the majority. Other layers are activation and pooling layers as shown in Figure 2.

#### 3.1.1. Basic CNN

Basic CNN is a repeated series connection of a *convolutional* layer, then an *activation* layer before a *pooling* or *downscaling* layer. Each set of layers detects an increasingly higher level of features as we move from the first layer to the *n-th* layer in the network. There are no branching of networks in a basic CNN configuration.

#### 3.1.2. VGG19-Frozen

VGG-19 [16] is a CNN with a special architectural modifications from its predecessors. It is a pre-trained neural network that won a top machine learning competition. Because it contains some high-level features of an images, not all layers will need to be modified when designing a new application. Hence, some layers can be frozen. Freezing a layer means that its weights cannot be modified by a new training algorithm. Only the layers that need to learn the distinguishing details of the new data will need to be modified. Freezing a layer is more beneficial for training than inference. This is because it reduces both training time and computational requirement as backward propagation does not transcend all the layers of the network. Various adapted freeze out techniques exist [17].

#### 3.1.3. VGG19-Fine Tuned

Fine-tuning involves the use of a base model and modifying training parameters to achieve better results on a new training set. Here, we employed the original VGG-19 as the pre-trained model. We then modify the model parameters to achieve the malaria classification problem.

#### 3.1.4. Mobile Net V2

This is a lightweight CNN model architecture that targets machine learning deployment into mobile devices [18]. The goal of the MobileNet model is to optimise compute power and memory.

### 3.2. Compression Methods and Prediction Accuracy

Achieving energy efficiency, memory management and fast inference time is important for edge devices especially in the developing communities of sub-saharan Africa. However, the prediction accuracy of the model should also be considered after compression of the model.

#### 3.2.1. Non-Converted (NC)

This is the model in its original form after training. It is not converted to a mobile version of any kind, and it not compressed as well. Hence, there is no memory optimisation after training.

#### 3.2.2. No Quantisation (NQ)

This is converted to Tensor Flow lite but neither the weights nor the activations are further quantised. The model weights and activations are normally stored with 32-bit precision. Unlike the NC, the NQ models have some levels of memory optimisation.

#### 3.2.3. Integer 8-Bit Quantisation of Weights Only (INT8WO)

These model weights are quantised to 8-bit precision. It is expected to have lower memory usage. Reduction from 32-bit to 8-bit is expected to achieve 4X memory reduction, which is beneficial for mobile devices used by health workers.

#### 3.2.4. Float16 Quantisation of Weights Only (Float16WOnly)

This is a 2X reduction in model size based on the weights only.

#### 3.2.5. Float 16 Quantisation of Both Weights and Activations (Float16WAndA)

This quantisation method reduces the precision of both the weights and the model activations.

The major aim of quantisation methods is to reduce memory size. However, we need to evaluate how this reduction impacts accuracy and inference time.

### 3.3. Mobile App Development

The malaria detection model with the best trade-off for a given environment is integrated into a mobile app. To be energy efficient, our model must be small enough to fit within the users’ target device memory alongside the rest of other programs. In resource depleted energy starved regions, mobile phones are the primary technology for the majority of peoples’ lives. To implement our model in an edge device, our target for energy efficiency will consider the memory sizes required for each architectures using training results shown in Figures 5–8, comprising Basic CNN, VGG19 frozen, VGG19 fine-tuned, and MobileNetV2, and then use TensorFlow Lite for post training model compression. The compressed model will be deployed on an Android device. TensorFlow Lite has suitable architecture designed for deployment in edge devices that will fit in memory without overly increasing processor workload and inference time [19]. Inference is performed using the TensorFlow Lite Java API and the TensorFlow lite Android support libraries. TensorFlow Lite has a built-in Benchmark App with tools that measure and calculate statistics to gauge performance metrics such as initialization, inference time, memory usage and confidence score [20]. The confidence score is a number between 0 and 1 that indicates how genuine a detection is relative to others. Based on Figures 6–8, our cut-off is more comfortable with false positives, images that were wrongly identified, or areas of the image that are erroneously identified as having the parasite when they are not and vice versa were cut-off. Thus, false negatives in which genuine images are misrepresented and missed will be low. We recommend placing the camera 10–12 cm directly over the image for optimal focus and capturing. We focus mostly on the inference speed and confidence values as a measure to validate that the algorithm has good specificity and energy savings. A high confidence value means a higher sort-able score for good recognition relative to others.

### 3.4. Case Finding with an AI-Enabled Mobile App

**Figure 3:**
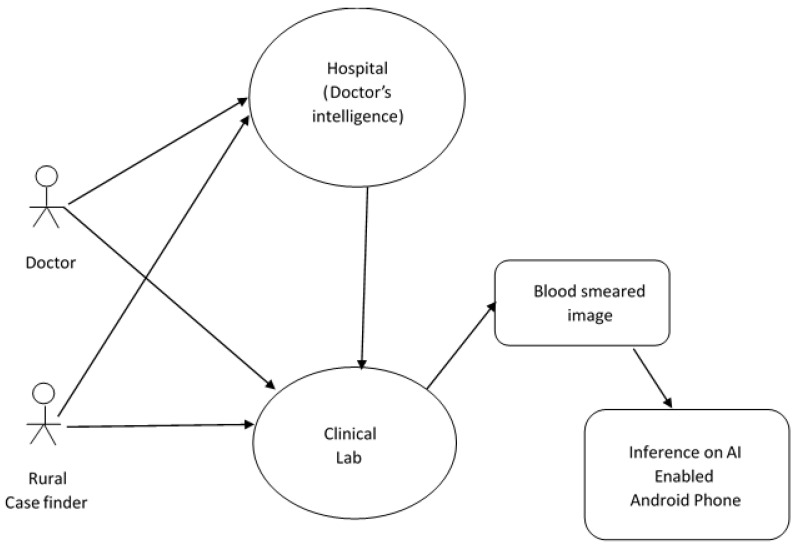
Use case for a typical local case finder The original expertise of thousands of medical doctors and lab technicians are contained in the original data set used to train machine learning models which less educated rural case finders can utilise for diagnosis without having the actual intelligence of medical doctors and lab technicians.

It is notable that global smartphone penetration continues to increase; as a result, our case finding demonstration was tested on smartphone brands, which accounted for over 66 percent of smartphones used in the continent [21], among which are OPPO, and Samsung, with a minimum of 2 GB RAM. Our case finding focused on memory usage by considering how long it takes to arrive at an interference and the confidence score of each prediction. We used pre-processed blood smeared images from the test set for optimization and validation. Once the App is presented with a captured image, it displays a visualization result on the phone screen classifying the image as healthy, malaria or unknown, when it could not determine the observed image with precision. The confidence of classification and the inference time gives an indication of the statistical power of decision on the observed image. A rural case finder can start a session once a picture of a pre-processed blood smear is fed into our AI system for inference as shown in Figure 3. A smart phone fitted with our malaria app placed directly over the image at approximately 10–15 cm will display a visualization of the result page on the screen indicating if the observed image is affected by malaria or healthy with a metric indicating the confidence level and inference time. A high confidence level is a measure of the degree of certainty with which our algorithm can rightly classify an observed image, ensuring that only positive cases are classified as being truly positive.

## 4. Results

We present our results starting from metrics that relate to our model application efficiency and then to metrics that ensure accurate detection of positive case of malaria on an edge device. Our simulation metrics focused on memory requirement, latency, prediction accuracy, recall and precision.

### 4.1. Assessment Metrics

#### 4.1.1. Memory Requirement at Different Levels of Quantization

**Figure 4:**
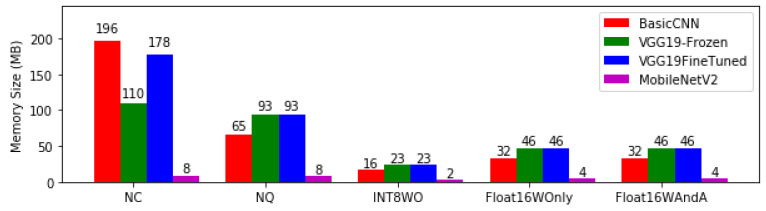
Memory size of model architectures at different levels of quantisation.

Figure 4 presents the model size of each of the model architectures and their respective quantisation levels, from non-converted (NC) to conversion with Float 16 quantisation of both weights and activations (Float16WAndA). Basic CNN and VGCFineTuned required approximately 200 MB of memory followed by VGG-19 Frozen while MobileNETV2 required only 8 MB. However, after conversion to TensorFlow Lite, Basic CNN dropped by 67%, VGGFineTunned 48% and VGG Frozen 15% while MobileNETV2 remained unchanged with the smallest memory usage and continued to maintain the lead in memory conservation for higher levels of quantization such as Float 16 weights only and Float 16 with weight and activation followed by basic CNN.

#### 4.1.2. Latency at Different Levels of Quantization

**Figure 5:**
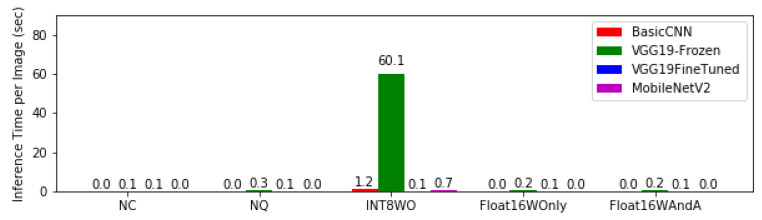
Latency or inference time for model architectures at different levels of quantisation.

Figure 5 shows the latency or inference time for model architectures at different levels of quantisation. Again, the inference time per image showed that the highest delay occurred with quantization with INT8WO; in addition, on average, each the model made an inference on an image in less than a second.

#### 4.1.3. Prediction Accuracy at Different Levels of Quantization

**Figure 6:**
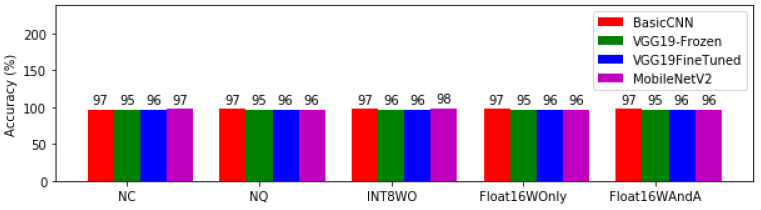
Prediction accuracy for model architectures at different levels of quantisation.

Figure 6 Is the prediction accuracy of each model architecture at different levels of quantisation. The maximum accuracy was 98% with MobileNETV2 at INT8WO level. On the other hand, the minimum accuracy was 95% with VGG-Frozen across all levels of quantization except INT8WO. Basic CNN was steady at 97% accuracy in all the tested levels of quantization.

#### 4.1.4. Recall at Different Levels of Quantization

**Figure 7:**
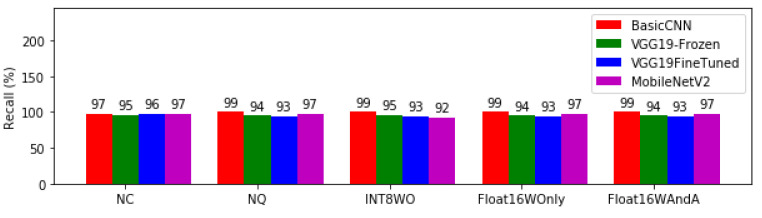
Recall for model architectures at different levels of quantisation.

The result shows that all our trained models have recall ranging from 92% to 99% across all quantization levels. INT8WO has the least percentage capability for the two most promising models—BasicCNN and MobileNETV2. However, prior conversion (NC) recall capability for both remained 97% and changed dramatically after conversion (NQ), with BasicCNN rising to 99% while MobileNetV2 dropped at Integer8 quantization with weight only (INT8WO). VGG19-Frozen and VGG19FineTuned had the weakest recall capability. Figure 7 depicts the recall for model architectures at different levels of quantization.

#### 4.1.5. Precision of Model Architecture at Different Levels of Quantization

**Figure 8:**
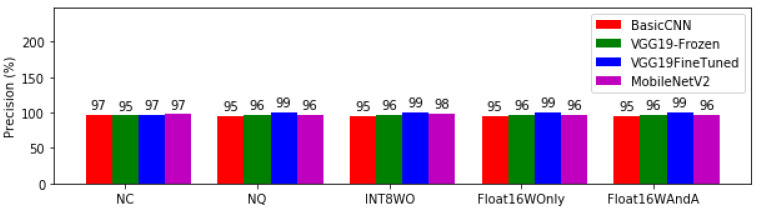
Precision of model architectures at different levels of quantisation.

The results for the percentage precision of the four models across the various levels of quantization showed VGGFineTuned leading by 99% after conversion. MobileNetV2 trailed VGGFineTuned with 98% precision, but dropped with increasing quantization of both weights and activations. BasicCNN has slightly lower precision than MobileNETV2 in all the different levels of quantization tested as shown in Figure 8.

### 4.2. Test Results for Mobile App

Table 1 shows the test results on two low-end commonly used Android smart phones with different storage and memory capacities. Three performance test results were shown: Classification, Inference time, and Confidence level. Appendix A is the image captured during testing. Samsung Galaxy A11 with 2 GB RAM and 32 GB storage space had a maximum inference time of 98 ms. Thus, we validate our model using inference time and confidence levels.

## 5. Discussion

Our results show the design trade-offs for a machine learning application to support malaria case finding in remote areas. Our bench marks were based on remarks that battery life for smart phones are constrained by CPU usage, device size, and the associated Network [22,23,24]. Therefore, we evaluated performance measures that affect the survival of mobile phone batteries in remote areas with memory size, latency, and performance parameters that relate to accurate detection of malaria cases on edge devices with precision, recall and prediction accuracy.

High recall (sensitivity) and high precision can be used together to ensure that people who have malaria are accurately identified. They can be utilised for both malaria suppression and elimination strategies. High recall is most useful in areas with a high prevalence of malaria in which the target is to reduce the number of transmissions. For regions that have low prevalence and low transmission, models with high precision will ensure that most people predicted as healthy are actually healthy [6]. The combination of high sensitivity and high precision models will be employed for aggressive malaria elimination in sub-regions where it is achievable due to low transmissions. The expressions for Precision, Recall, Sensitivity and Specificity can be found in [6,7]. From the results, we make the following deductions:

### 5.1. Model Comparisons

#### 5.1.1. Memory Size and Latency of Models

MobileNetV2 has the smallest memory requirement for all levels of quantisation. For other models, the level of quantisation determines the memory consumption. Overall, Basic CNN has the largest memory requirement when not converted (NC) to the mobile version of the model. However, when converted to mobile format but not quantised (NC), VGG-19 models would have higher memory requirements than Basic CNN. This reduction in size for basic CNN remains the same for all other levels of quantisation. Hence, apart from MobileNetV2, basic CNN is the next model to be considered when a quantised model is to be deployed in a mobile device if optimised memory is the major design goal. Since CPU utilization is related to power consumption [22,23], MobilNETV2 has higher architectural improvement by energy savings.

When considering latency and speed, MobileNetV2 still has the best average performance across all levels of quantisation. Although its performance for INT8WO is worse than that of VGG-19Finetuned, MobileNetV2 consistently has fast inference time across other quantisation levels. Basic CNN network architecture has the next optimum performance if we consider INT8WO as an outlier in terms of speed. It can be clearly seen from Figure 5 that INT8WO quantisation for all models took a significant amount of time than any other quantisation. Having said this, one can see that the inference time for VGG-19Frozen when performed with INT8W0 quantisation is significantly high and cannot be considered for mobile case finding.

Hence, when it comes to memory optimisation, MobileNetV2 with INT8WO quantisation is the best. However, for speed, the INT8WO is not as fast as Float16WOnly or Float16WAndA. Hence, if inference speed is a design goal, any of the MobileNetV2 or Basic CNN architecture will be chosen provided that INT8WO quantisation is not applied to the model. To optimise both speed and latency, only MobileNetV2 with either Float16WOnly or Float16WAndA quantisation should be considered.

#### 5.1.2. Diagnostic Performance

The diagnostic performance of medical diagnostic systems is measured in terms of sensitivity and specificity. However, in machine learning terms, we talk about accuracy, recall and precision. Figure 6 shows the accuracy of the models for each level of quantization.

The best accuracy of 98% belongs to MobileNetV2 with INT8WO quantisation. It is noticed that VGG19Frozen has the worst accuracy across all quantisation levels. This achievement is another good score to MobileNetV2 with INT8WO quantisation as it previously emerged as the model with the least memory requirement. Second to MobileNetV2 in terms of accuracy is Basic CNN. It has an accuracy of 97% for all quantisation levels. This steady average performance is comparable to MobileNetV2 that varied from 96% to 98% accuracy across all quantisation levels.

Recall and Precision are considered better performance metrics than accuracy especially for unbalanced datasets. Recall or sensitivity is also a very important measure in healthcare machine learning as it defines the confidence of an AI system in ensuring that only positive cases are classified as being truly positive. High recall reduces false negatives [7].

Basic CNN (INT8WO, Float16WOnly and Float16WAndA) has the best recall of 99% in almost all quantisation levels as can be seen in Figure 7. If this model is deployed into the mobile application used for active case finding in remote areas, out of 100 tests, only one false alarm would be experienced. High sensitivity ensures that most diseases that present in a way similar to malaria will be differentiated with high accuracy and will not be confused with patients who actually have malaria. After Basic CNN, the next model to be considered is MobileNetV2 with 97% sensitivity. For all levels of model quantisation, all the VGG-19 models have lower recall than both Basic CNN and MobileNetV2. Considering previous metrics that we have examined, Basic CNN and MobileNetV2 maintain a higher performance. We examine model precision next.

All converted (NQ) and quantised versions (INT8WO, Float16WOnly and Float16WAndA) of VGG19Finetuned have the highest precision of 99% (see Figure 8). Precision relates to Type I error or false positives. A precision of 99% means that, out of 100 people detected as healthy, only 99 people are healthy, while one person that was detected as being healthy probably has malaria. Hence, if the purpose of the deep learning model is to exclude people who have malaria, VGG19Finetuned would be the best model to deploy.

Models with high precision will have low recall and vice versa. VGG19Finetuned INT8WO with 99% precision has 93% recall while Basic CNN INT8WO with 95% precision has 99% recall. If only one model could be deployed into a mobile application, then Basic CNN models have higher average performance on recall and precision than VGG19Finetuned INT8WO and thus should be deployed. However, the results here show that the potential for the two models could help in verifying test results by using the two models in a series depending on malaria prevalence within a region.

The short inference time with a high confidence score proved that the system rightly classifies malaria and healthy individuals when used on a pre-processed smeared blood image. It also demonstrated effective improvement for energy savings when deployed on Android enabled smart phones with low memories.

## 6. Conclusions

In this paper, we evaluated the design trade-offs for a machine learning model to support malaria case finding in remote and resource-constrained areas. We considered performance metrics that affect the battery life of mobile phone batteries in remote areas (memory size and latency) and performance parameters that relate to accurate detection of malaria cases using machine learning models adapted for deployment in edge devices. We discussed various scenarios for which each model can be applied and demonstrated a typical case finding using Android phones typical in the regions of concern. We showed that incorporating quantised deep machine learning models into a mobile application can be used for effective active malaria case finding in areas with high prevalence of malaria infection.

In our future work, we will explore a clinical trial that demonstrates positive predictive values of our model in verifying its efficacy to rightly discriminate persons with malaria from those without disease and from ambiguously smeared blood images. We will also examine how our system can categorize the severity of each sensitive case by integrating specific indices of the blood smear, such as parasite count, into our model.

## Figures and Tables

**Table 1:** Test results on mobile App.

Device	Classification	Inference Time (ms)	Confidence Estimate
Oppo3, Android 11,			
Storage 64 GB,			
RAM-4.0GB			
	Healthy	34	100
	Malaria	34	100
Samsung Galaxy A11,			
Android 10,			
Storage 32 GB,			
RAM-2.0 GB			
	Healthy	97	100
	Malaria	98	100

**Figure A1:**
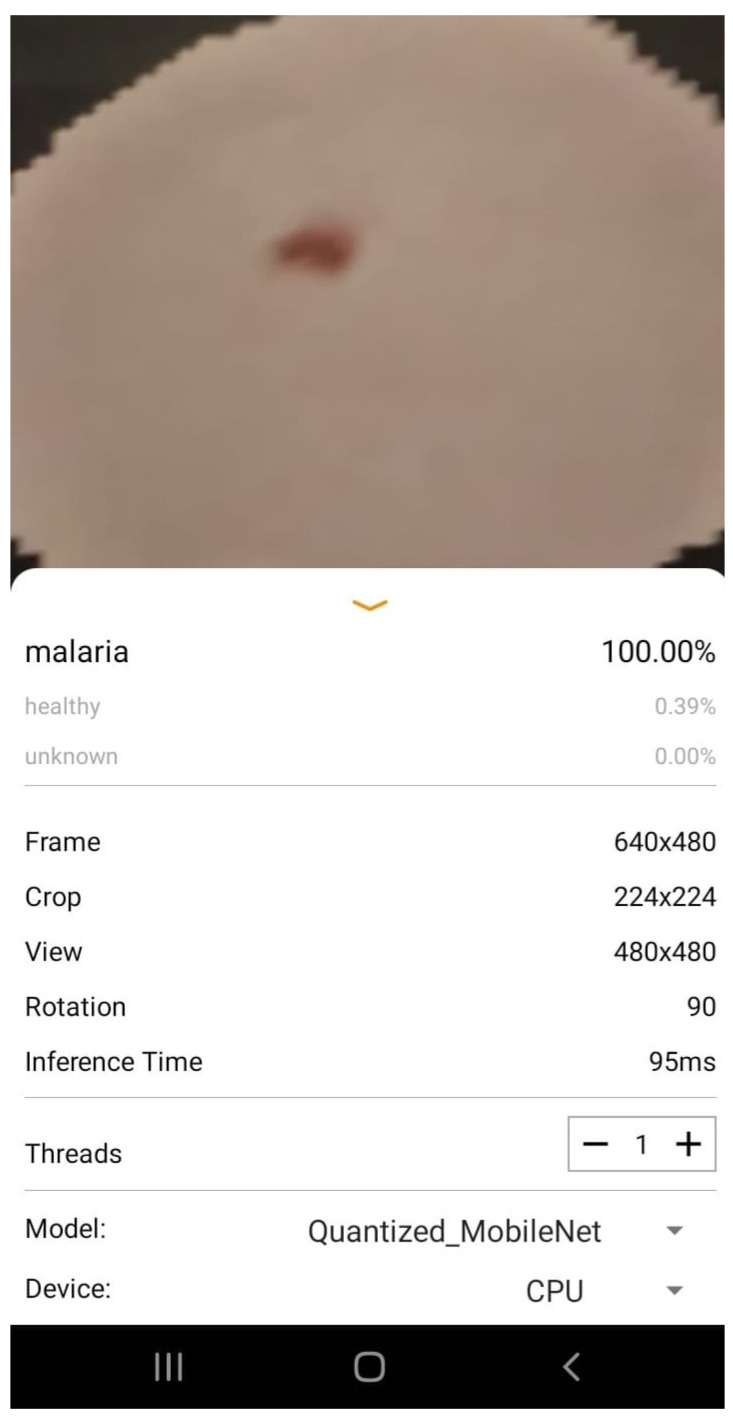
Malaria classified image on a Samsung Galaxy A11 running Android 10.

**Figure A2:**
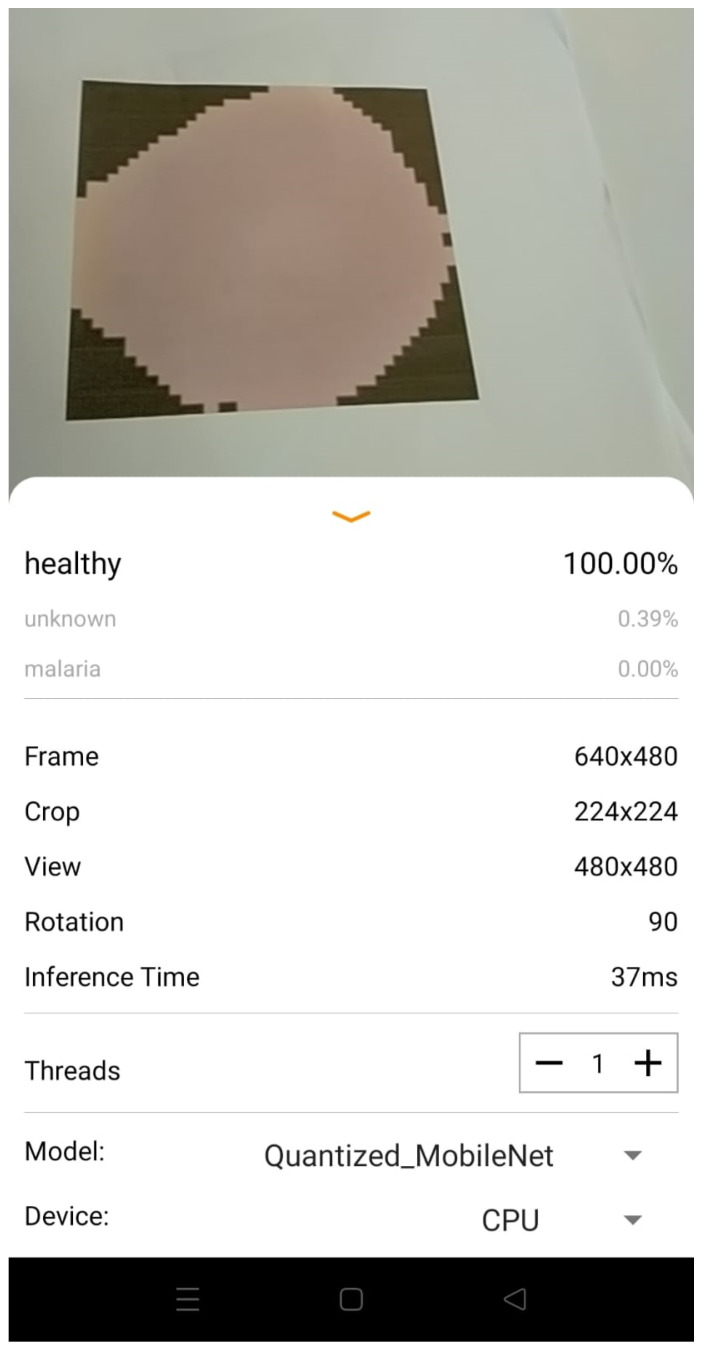
Healthy classified image on an Oppo3 running Android 11.

## Data Availability

Kaggle. Malaria Parasite Detection: Parasite Detection in Thin Blood Smear Image. 2020. Available online: https://www.kaggle.com/c/malaria-parasite-detection/data.

## Appendix A. Mobile Application Execution

The screenshots below show the test result when the MobileNetV2 is used for malaria detection using the image of a blood smear. We used samples with malaria (Figure A1), healthy (Figure A2) image types. Each class was detected with 100% accuracy.
